# Supplementary material for: Plasma Ag-Modified α-Fe2O3/g-C3N4 Self-Assembled S-Scheme Heterojunctions with Enhanced Photothermal-Photocatalytic-Fenton Performances
Source: Nanomaterials (Basel). 2022 Nov 27;12(23):4212. doi: 10.3390/nano12234212 (PMC9740289; doi:10.3390/nano12234212)
Supplement: Supplementary file 1 [file nanomaterials-12-04212-s001.zip › nanomaterials-2050077-supplementary.pdf]

nanomaterials

*Supplementary Material*

# Plasma Ag-Modified α-$Fe_2O_3$/g-$C_3N_4$ Self-Assembled S-Scheme Heterojunctions with Enhanced Photothermal-Photocatalytic-Fenton Performances

**Yawei Xiao [1], Bo Yao [1], Zhezhe Wang [1], Ting Chen [2,*], Xuechun Xiao [1,*] and Yude Wang [3,*]**

[1] National Center for International Research on Photoelectric and Energy Materials, School of Materials and Energy, Yunnan University, Kunming 6500504, China
[2] Institute of Materials Science & Devices, School of Materials Science and Engineering, Suzhou University of Science and Technology, Suzhou, 215009, China
[3] Yunnan Key Laboratory of Carbon Neutrality and Green Low-Carbon Technologies, Yunnan University, Kunming 6500504, China
* Correspondence: chenting@mail.usts.edu.cn (T.C.); xchxiao@ynu.edu.cn (X.X.); ydwang@ynu.edu.cn (Y.W.)

## Materials

Melamine ($C_3H_6N_6$, AR, ≥99.9%), Cyanuric acid ($C_3H_3N_3O_3$, AR, ≥99.9%), Iron(III) chloride hexahydrate ($FeCl_3·6H_2O$, AR, ≥99.9%), Silver nitrate ($AgNO_3$, AR, ≥99.9%), Sodium acetate ($CH_3COONa$, AR, ≥99.9%) were purchased from Aladdin Reagent (Shanghai) Co. Ltd, China. Ethanol absolute ($C_2H_6O$, ≥99.5%) was purchased from Shanghai Maclean Biochemical Technology Co. Ltd, China. All chemicals were sourced from commercial suppliers and were not further purified before being used, and deionized water was used throughout the experiments.

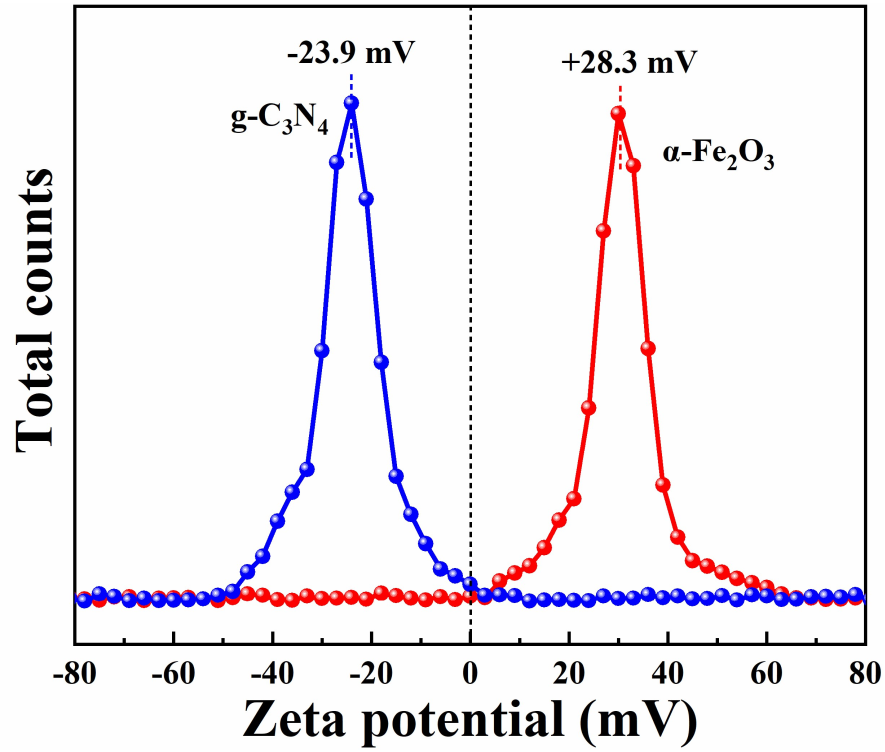


**Figure S1.** Zeta potential of the g-$C_3N_4$ and acidified α-$Fe_2O_3$ dispersed in deionized water at pH 7.

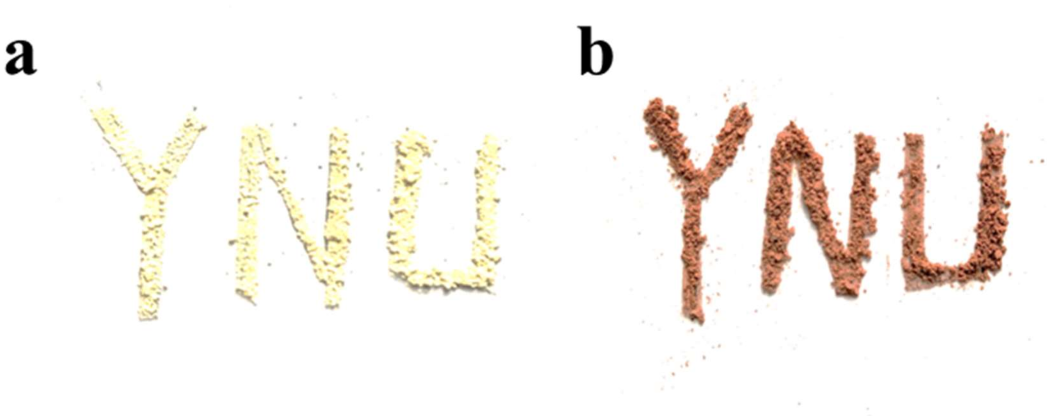


**Figure S2.** The sample patterns of g-$C_3N_4$ and Ag/$\alpha$-$Fe_2O_3$/g-$C_3N_4$.

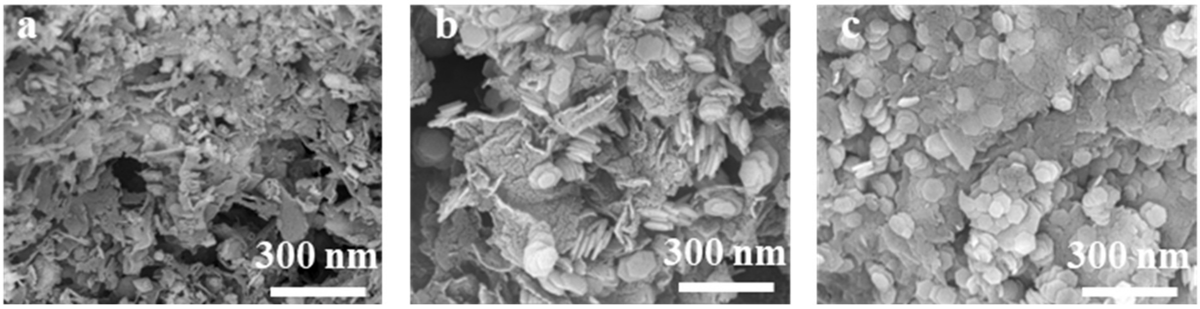


**Figure S3.** SEM images of $\alpha$-$Fe_2O_3$/g-$C_3N_4$ for $\alpha$-$Fe_2O_3$ weight ratios 20% (a), 30% (b), 40% (c).

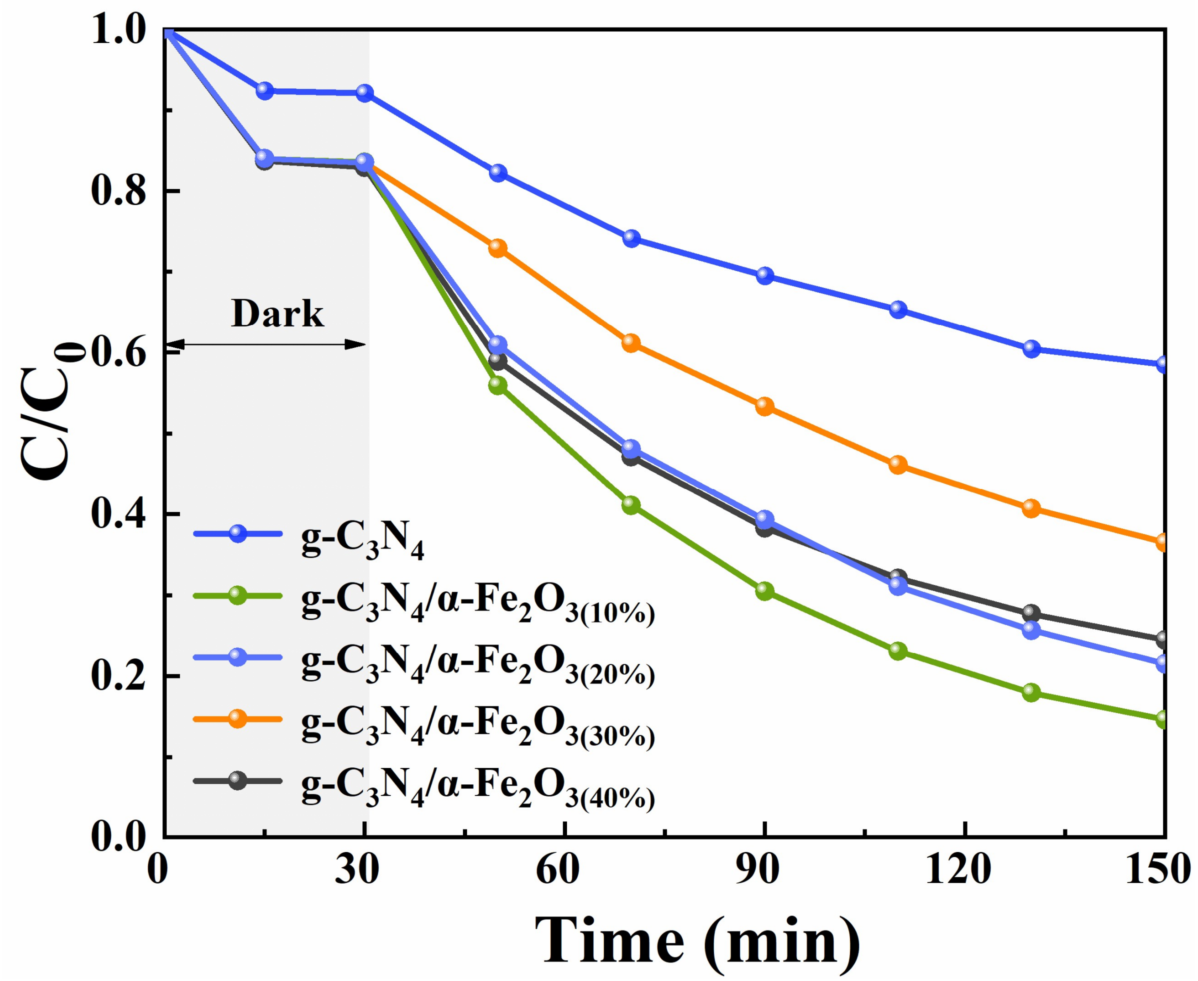


**Figure S4.** Degradation of TC by different weight ratios of $\alpha$-$Fe_2O_3$/g-$C_3N_4$.

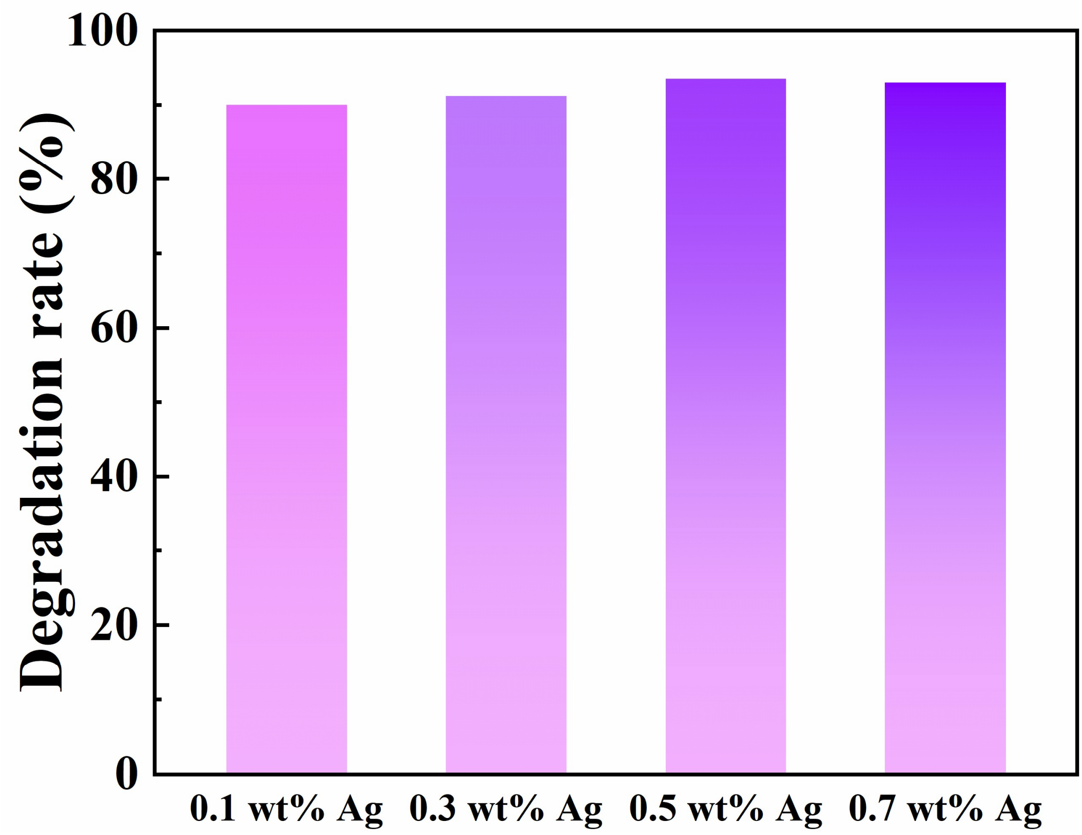


**Figure S5.** Degradation of TC by catalysts with different Ag loadings.

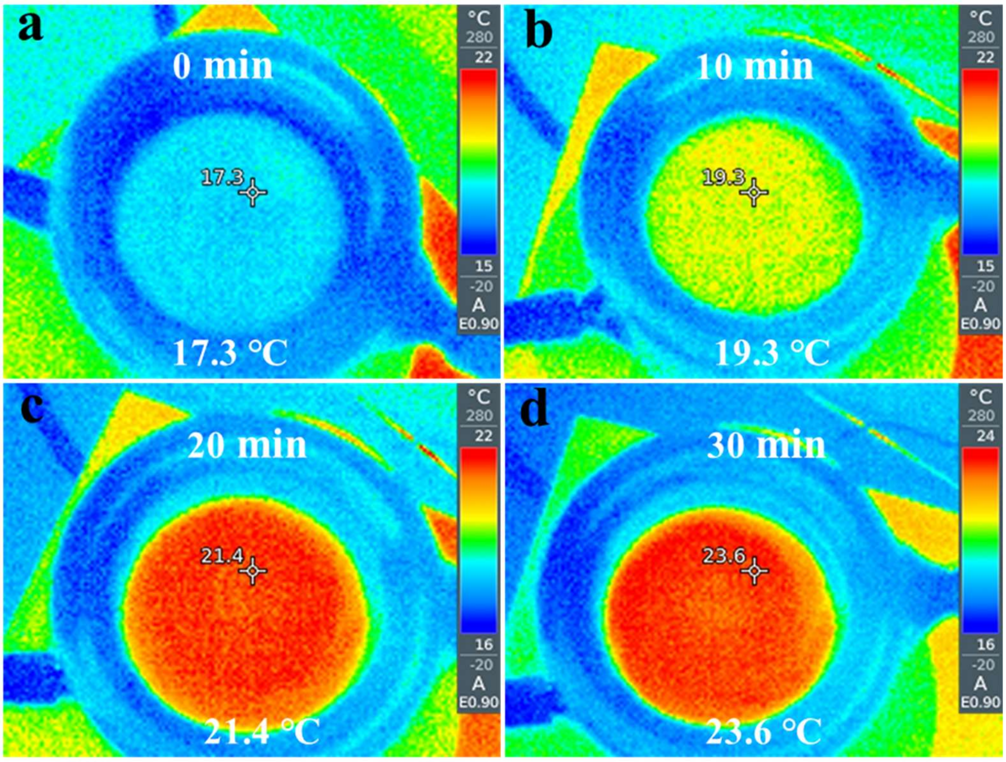


**Figure S6.** IR images of the pure water under illumination with a 300 w xenon lamp as a function of time.

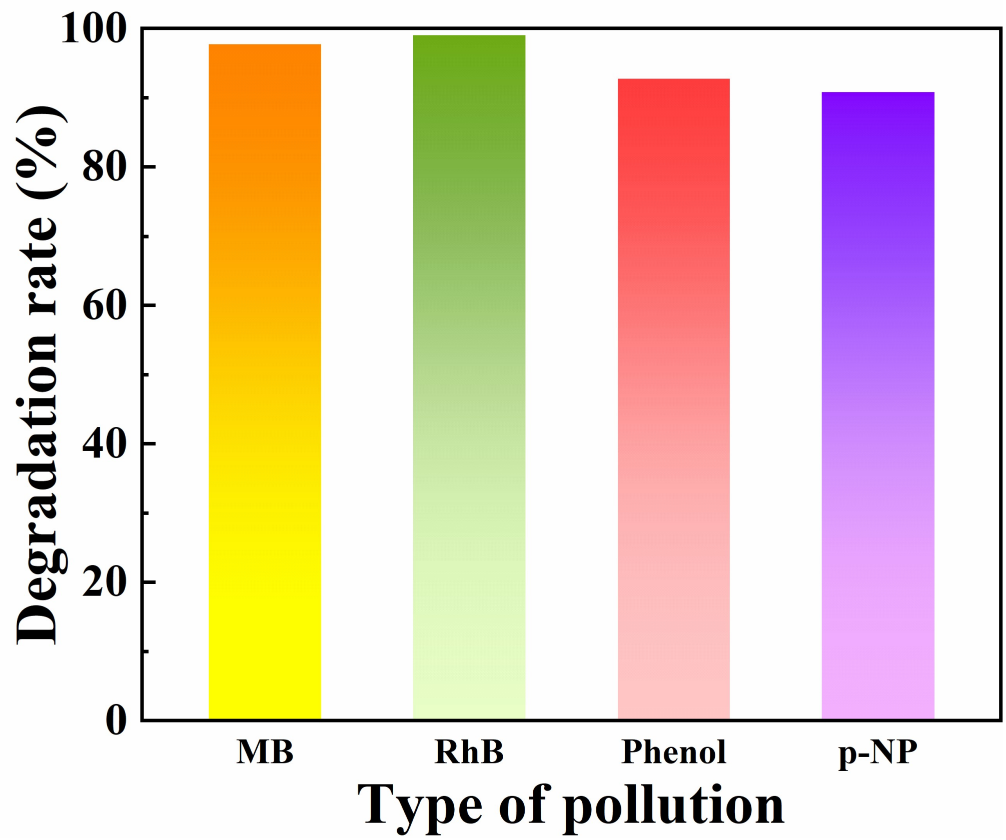


**Figure S7.** Photocatalytic degradation of other pollutants by Ag/$\alpha$-$Fe_2O_3$/g-$C_3N_4$.

**Table S1.** Comparison of the performance of Ag/$\alpha$-$Fe_2O_3$/g-$C_3N_4$ and other similar photocatalysts for $H_2$ evolution.

| Photocatalysts | Light source | $H_2$ evolution rate (μmol $g^{-1}$ $h^{-1}$) | Ref. |
|---|---|---|---|
| **Ag/$\alpha$-$Fe_2O_3$/g-$C_3N_4$** | **Xe lamp (300W)** | **3125** | **This work** |
| $MoS_2$/CdS/g-$C_3N_4$ | 300 W Xe lamp ($\lambda$>420 nm) | 956 | [8] |
| KCCN | Xe lamp ($\lambda$>420 nm) | 557 | [9] |
| $\alpha$-$Fe_2O_3$/CdS/g-$C_3N_4$ | Xe lamp (1000 W) | 165 | [10] |
| $\alpha$-$Fe_2O_3$/g-$C_3N_4$(Pt) | Xe lamp (300W, $\lambda$>420 nm) | 5000 | [11] |
| $ZnIn_2S_4$/$Ti_3C_2$ | Xe lamp (300 W, $\lambda$>420 nm) | 979 | [12] |
| g-$C_3N_4$/$WO_3$-carbon microsphere | Xe lamp (300W, $\lambda$ > 420 nm) | 2500 | [13] |
| hollow core-shell $TiO_2$/g-$C_3N_4$ | Xe lamp (300 w) | 809 | [14] |

**Table S2.** Comparison of the photocatalytic performance of Ag/α-$Fe_2O_3$/g-$C_3N_4$ and other similar photocatalysts for TC degradation.

| Photocatalysts | Light source | TC Removal rate | Ref. |
|---|---|---|---|
| **Ag/α-$Fe_2O_3$/g-$C_3N_4$** | **Xe lamp (300W, λ>420 nm)** | **93.6%** | **This work** |
| $TiO_2$-P25 | Xe lamp (300 W, λ=350 nm) | 94.8% | [1] |
| $CuInS_2/Bi_2MoO_6$ | Xe lamp (300 W, λ>420 nm) | 84.7% | [2] |
| $Ag_2CO_3/Bi_4O_5I_2$/g-$C_3N_4$ | Xe lamp (300 W, λ>420 nm) | 82.2% | [3] |
| β-$Bi_2O_3$@g-$C_3N_4$ | Xe lamp (250 W, λ>420 nm) | 80.2% | [4] |
| g-$C_3N_4/ZrO_{2-x}$ NTs | Xe lamp (300 W, λ>420 nm) | 90.6% | [5] |
| CDs/g$C_3N_4/MoO_3$ | Xe lamp (350W, λ > 420 nm) | 88.4% | [6] |
| g-$C_3N_4$/carbon nanotubes/$Bi_{25}FeO_{40}$ | Xe lamp (500 w, λ > 420 nm) | 87.9% | [7] |

## References


[1] Wu, S.; Hu, H.; Lin, Y.; Zhang, J; Hu. Y Visible light photocatalytic degradation of tetracycline over $TiO_2$. Chem. Eng. J. **2020**, 382, 122842.

[2] Guo, J.; Wang, L.; Wei, X.; Alothman, Z.; Albaqami, M.; Malgras, V.; Yamauchi, Y.; Kang, Y.; Wang, M.; Guan, W.; Xu, X. Direct Z-scheme $CuInS_2/Bi_2MoO_6$ heterostructure for enhanced photocatalytic degradation of tetracycline under visible light. J. Hazard. Mater. **2021**, 415, 125591.

[3] Chen, Z.; Guo, H.; Liu, H.; Niu, C.; Huang, D.; Yang, Y.; Liang, C.; Li, L.; Li, J. Construction of dual S-scheme $Ag_2CO_3/Bi_4O_5I_2$/g-$C_3N_4$ heterostructure photocatalyst with enhanced visible-light photocatalytic degradation for tetracycline. Chem. Eng. J. **2022**, 438, 135471.

[4] Hong, Y.; Li, C.; Yin, B.; Li, D.; Zhang, Z.; Mao, B.; Fan, W.; Gu, W.; Shi, W. Promoting visible-light-induced photocatalytic degradation of tetracycline by an efficient and stable beta-$Bi_2O_3$@g-$C_3N_4$ core/shell nanocomposite. Chem. Eng. J. **2018**, 338, 137-146.

[5] Chen, Q.; Yang, W.; Zhu, J.; Fu, L.; Li, D.; Zhou, L. Enhanced visible light photocatalytic activity of g-$C_3N_4$ decorated $ZrO_{2-x}$ nanotubes heterostructure for degradation of tetracycline hydrochloride. J. Hazard. Mater. **2020**, 384, 121275.

[6] Xie, Z.; Feng, Y.; Wang, F.; Chen, D.; Zhang, Q.; Zeng, Y.; Lv, W.; Liu, G. Construction of carbon dots modified $MoO_3$/g-$C_3N_4$ Z-scheme photocatalyst with enhanced visible-light photocatalytic activity for the degradation of tetracycline. Appl. Catal. B: Environ. **2018**, 229, 96-104.

[7] Li, X.; Qiu, Y.; Zhu, Z.; Zhang, H.; Yin D. Novel recyclable Z-scheme g-$C_3N_4$/carbon nanotubes/$Bi_{25}FeO_{40}$ heterostructure with enhanced visible-light photocatalytic performance towards tetracycline degradation. Chem. Eng. J. **2022**, 429, 132130.

[8] Zhao, T.; Xing, Z.; Xiu, Z.; Li, Z.; Yang, S.; Zhou, W, Oxygen-doped $MoS_2$ nanospheres/CdS quantum dots/g-$C_3N_4$ nanosheets super-architectures for prolonged charge lifetime and enhanced visible-light-driven photocatalytic performance, ACS Appl. Mater. Interfaces, **2019**, 11, 7104-7111.

[9] Zhang, G.; Xu, Y.; Yan, D.; He, C.; Li, Y.; Ren, X.; Zhang, P.; Mi, H. Construction of $k^+$ ion gradient in crystalline carbon nitride to accelerate exciton dissociation and charge separation for visible light $H_2$ production, ACS Catal. **2021**, 11, 6995-7005.

[10] Yavuz, C.; Erten-Ela, S. Solar light-responsive $\alpha$-$Fe_2O_3$/CdS/g-$C_3N_4$ ternary photocatalyst for photocatalytic hydrogen production and photodegradation of methylene blue. J. Alloys Compd. **2022**, 908, 164584.

[11] Li, Y.; Zhu, S.; Liang, Y.; Li, Z.; Wu, S.; Chang, C.; Luo, S.; Cui, Z. Synthesis of $\alpha$-$Fe_2O_3$/g-$C_3N_4$ photocatalyst for high-efficiency water splitting under full light. Mater. Des. **2020**, 196, 109191.

[12] Huang, W.; Li, Z.; Wu, C.; Zhang, H.; Sun, J.; Li, Q. Delaminating $Ti_3C_2$ MXene by blossom of $ZnIn_2S_4$ microflowers for noble-metal-free photocatalytic hydrogen production. J Mater Sci Technol **2022**, 120, 89-98.

[13] Sun, M.; Zhou, Y.; Yu, T.; Wang J. Synthesis of g-$C_3N_4$/$WO_3$-carbon microsphere composites for photocatalytic hydrogen production. Int. J. Hydrog. Energy **2022**, 47, 10261-10276.

[14] Pan, J.; Dong, Z.; Wang, B.; Jiang, Z.; Zhao, C.; Wang, J.; Song, C.; Zheng, Y.; Li, C. The enhancement of photocatalytic hydrogen production via $Ti^{3+}$ self-doping black $TiO_2$/g-$C_3N_4$ hollow core-shell nano-heterojunction. Appl. Catal. B: Environ. **2019**, 242, 92-99.
